# P-973. Impact of an Antibiotic Stewardship Intervention on β-lactam Use in Orthopedic Surgery Patients with Beta-Lactam Allergy

**DOI:** 10.1093/ofid/ofaf695.1172

**Published:** 2026-01-11

**Authors:** Leen Dabbas, Eyad Sawalha, Sarah Graziose, Saumil Doshi

**Affiliations:** Medstar Washington Hospital Center, Washington, District of Columbia; University of Jordan, Washington, District of Columbia; Medstar Washington Hospital Center, Washington, District of Columbia; Medstar Washington Hospital Center, Washington, District of Columbia

## Abstract

**Background:**

Penicillin allergy labels are common but often inaccurate, resulting in suboptimal antibiotic use. PEN-FAST scoring can identify low-risk patients but remains underused in surgical settings. We assessed whether preoperative interviews and PEN-FAST scoring increased perioperative β-lactam use in orthopedic patients with reported penicillin allergy

**Figure:**
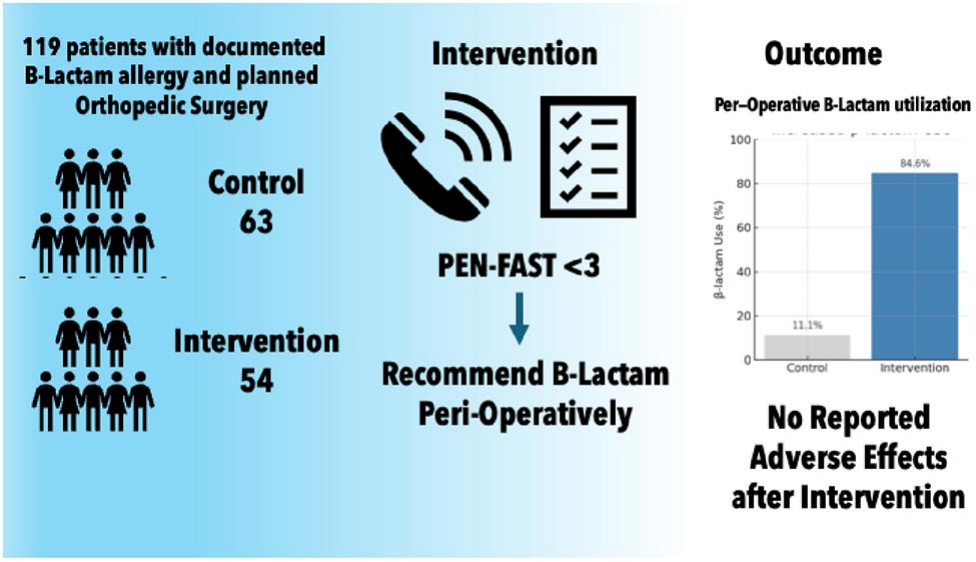

**Figure:**
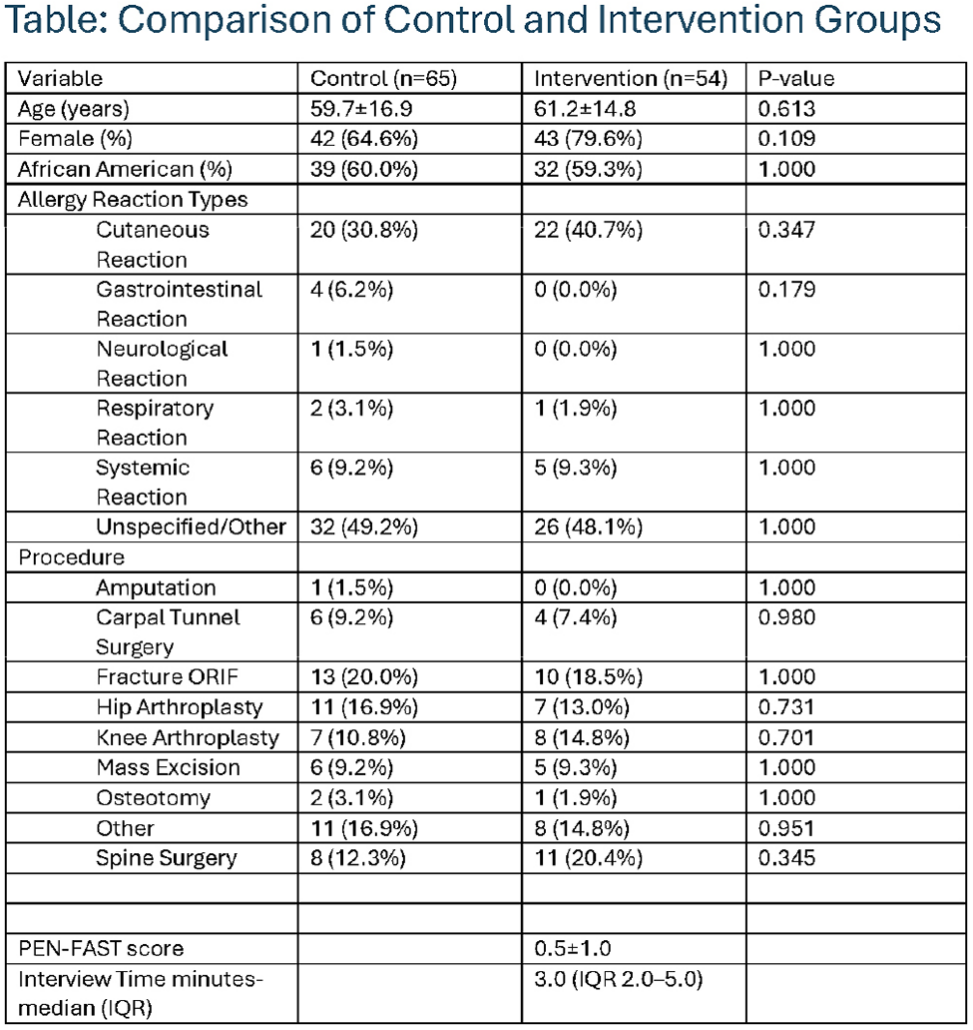

**Methods:**

Design: Quality improvement study at a single center. Participants: Adult surgical patients with a documented β-lactam allergy. The intervention group (Jan–Jul 2022) underwent preoperative interviews and PEN-FAST scoring. The control group (Jan–Jul 2021) did not receive the intervention. Intervention: Patients with a PEN-FAST score < 3 were identified as low risk, and the team contacted orthopedics to recommend β-lactam antibiotics. Outcome: Primary outcome was the proportion receiving β-lactams perioperatively. Secondary outcome was a composite safety endpoint (epinephrine or antihistamine use within 24 hours). Interview duration was recorded for feasibility.

**Results:**

A total of 119 patients were included (control: n=65; intervention: n=54). Groups were similar in age (59.7±16.9 vs. 61.2±14.8 p=0.61), race (60.0% vs. 59.3% African American p=1.00), and sex (79.6% vs. 64.6%, p=0.10). Hip or knee arthroplasty was the most common procedure (62% control vs. 59% intervention). Historical cutaneous reactions were most frequently reported (31% control vs. 41% intervention), with systemic reactions present in 9% of both groups. Nearly half of all patients had no documented reaction types. The mean PEN-FAST score in the intervention group was 0.5±1.0. Median interview time in the intervention group was 3.0 minutes (IQR: 2.0–5.0). β-lactam use was higher in the intervention group (84.6% vs 11.1%, p < 0.01). No patients in the intervention group required epinephrine or experienced anaphylaxis.

**Conclusion:**

A PEN-FAST–guided stewardship protocol incorporating patient interviews and proactive provider recommendations significantly increased perioperative β-lactam use without safety events. This approach is feasible, safe, and improves antibiotic selection in surgical patients with reported penicillin allergy.

**Disclosures:**

All Authors: No reported disclosures

